# Neoplastic plasma cells generate an inflammatory environment within bone marrow and markedly alter the distribution of T cells between lymphoid compartments

**DOI:** 10.18632/oncotarget.16628

**Published:** 2017-03-28

**Authors:** Oliver C. Goodyear, Sarah Essex, Anandram Seetharam, Supratik Basu, Paul Moss, Guy Pratt

**Affiliations:** ^1^ Institute of Immunology and Immunotherapy, College of Medical and Dental Sciences, University of Birmingham, Birmingham, UK; ^2^ Department of Haematology, The Royal Wolverhampton Hospitals NHS Trust, Wolverhampton, UK; ^3^ Birmingham Health Partners, Centre for Clinical Haematology, University Hospital Birmingham NHS Foundation Trust, Birmingham, UK

**Keywords:** myeloma, chemokine

## Abstract

Monoclonal gammopathy of undetermined significance (MGUS) and multiple myeloma (MM) are characterised by the accumulation of malignant plasma cells within bone marrow and lead to a range of abnormalities in the peripheral blood T cell repertoire. We investigated the level of inflammatory chemokines within the bone marrow and blood of patients with MGUS and MM and related this to the pattern of chemokine receptor expression on T cells in both compartments.

The expression of a wide range of chemokine ligands for CXCR3 and CCR4 was markedly increased within the bone marrow of patients with MGUS and MM compared to healthy donors. The most marked effects were seen for CCL4 and CXCL9 which were increased by 4 and 6 fold respectively in the bone marrow of patients with myeloma. The expression of CXCR3 and CCR4, the major TH1 and TH2-associated chemokine receptors, was increased substantially on T cells within the bone marrow of patients whereas the percentage of CXCR3-expressing T cells within blood was correspondingly decreased. The presence of even small numbers of neoplastic plasma cells or associated stroma can therefore generate an inflammatory chemokine tumour microenvironment. This leads to the selective recruitment or retention of specific T cell subsets which is likely to underlie many of the features regarding the peripheral T cell repertoire in myeloma and may also contribute to the immune suppression associated with this disease. This local inflammatory reaction may represent a tumour-specific immune response or may itself play an important role in tumour progression and as such may offers a potential novel target for therapeutic intervention.

## INTRODUCTION

Monoclonal gammopathy of undetermined significance (MGUS) and multiple myeloma (MM) are related disorders defined by the presence of neoplastic plasma cells within the bone marrow and monoclonal immunoglobulin within serum. MGUS is an asymptomatic premalignant form of myeloma with an approximately 1% risk each year of progression to MM for an individual patient. Indeed, it is now believed that MGUS precedes the development of virtually every case of MM [1, 2].

Patients with MM exhibit a variety of numerical and functional T cell abnormalities, including increased numbers of memory cells, reduced clonal diversity and impaired functional and viral-specific responses [3]. A relative accumulation of T cells within the bone marrow has also been reported and is associated with the development of an oligoclonal TCR-Vβ repertoire [4–7]. These changes may contribute to the relative immunodeficiency of MM but their aetiology and relevance to tumour progression is unknown. The role of regulatory T cells in the development of MM is also under debate and remains controversial [8–12]. T_H_17 cells are also of considerable interest in relation to tumour immunity and are increased in both the marrow and blood of patient with paraproteinaemia [13–23].

T cells in the bone marrow of patients with paraproteinemia might potentially be involved in either the suppression or support of the tumour cell population. Indeed, T_H_17-associated pro-inflammatory cytokines have been shown to increase the survival of MM cells both *in vitro* and *in vivo* [10, 13, 24, 25] and may play a role in development of bone disease [13, 26].

The altered distribution of T cell subsets between the bone marrow and peripheral blood of patients with paraproteinaemia is likely to reflect the pattern of migration and retention of cells within marrow. Chemokine gradients are of central importance and there are data showing the importance of an upregulated CXCR4/CXCR7/CXCL12 axis in driving recruitment of tumour cells [27–28] into the bone marrow in patients with multiple myeloma. Other chemokines have been shown to be elevated in patients with multiple myeloma including CCL3 and CCL20 [29–30]. Increased levels of key cytokines have also been shown to progressively increase from controls to smouldering myeloma to multiple myeloma including CXCL8 (IL-8), IL-10, TNFα, IL6 and IFNγ [31]. A decrease in NK cell numbers in the bone marrow of patients with myeloma has been shown to correlate with a decrease in the chemokine CXCL12 and an upregulation of CXCR3 ligand expression [32].

We therefore measured chemokine levels within these compartments and related these findings to the distribution of chemokine receptor expression on CD4+ and CD8+ T cells. Our findings reveal an inflammatory tumour microenvironment within the bone marrow in patients with paraproteinaemia that markedly alters the distribution of individual T cell subsets and leads to accumulation of T cells within the tumour microenvironment.

## RESULTS

### The expression of a wide range of inflammatory chemokines is increased in the bone marrow and blood of patients with paraproteinaemia

**Figure 1:**
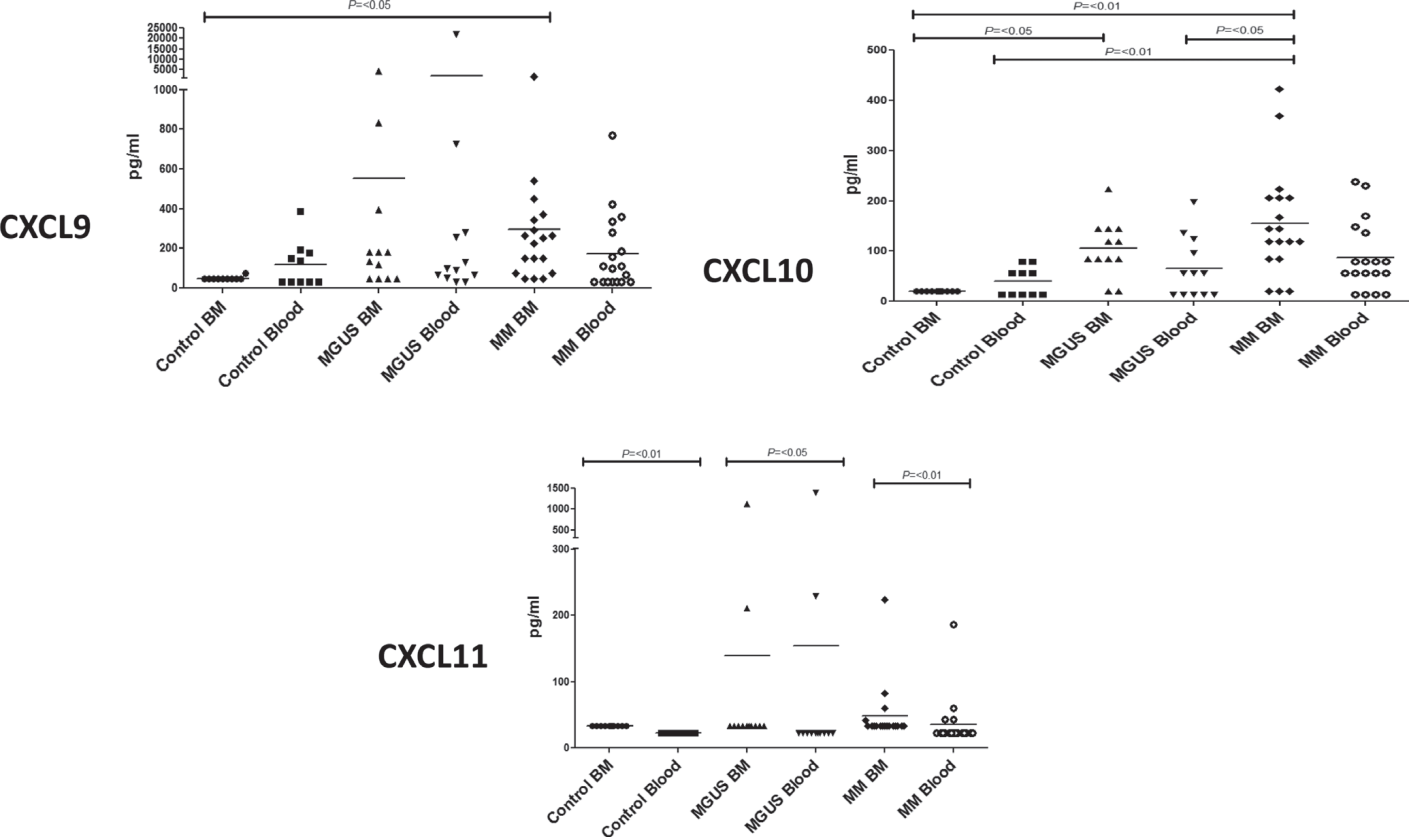
Plasma cytokine levels (pg/ml) of CXCR3 related ligands were measured in bone marrow (BM) and blood in MM, MGUS and control patients by multiplex bead analysis (A) CXCL9 (B) CXCL10 (C) CXCL11 Data were analysed using a Kruskal Wallis where *** = *p* < 0.001, ** = *p* < 0.01, *p < 0.05.

In initial studies we focussed on the level of expression of a range of inflammatory chemokines that are ligands for the chemokine receptors CXCR3 and CCR4. These two receptors were chosen as their expression is relatively selective on T_H_1 and T_H_2 cells respectively. CXCR3 is expressed on virtually all T_H_1 cells and binds to three chemokine ligands CXCL9 (MIG), CXCL10 (IP-10) and CXCL11 (ITAC). We used Luminex analysis to determine the pattern of expression of these ligands within marrow and blood samples from the study cohort (Figure 1A–1C).

All three chemokines were expressed at low levels in the control group. However, it was noteworthy that CXCL9 and CXCL10 were undetectable within the bone marrow whereas low levels were observed within blood. This indicates that the expression of these inflammatory chemokines is normally under tight regulation within the marrow compartment.

Interestingly, the expression of both CXCL9 and CXCL10 was markedly increased in the bone marrow of the patient groups, and appeared to increase in a progressive fashion during the clinical transition from MGUS to myeloma although a definitive longitudinal study would be needed to confirm this. Of note was the observation that expression was higher within the bone marrow than the blood which lead to a reversal of the normal marrow:blood gradient of chemokine expression (Figure 1A and 1B). No major or consistent changes were observed in relation to expression of CXCL11 between MM or MGUS patients and the control group (Figure 1C).

**Figure 2:**
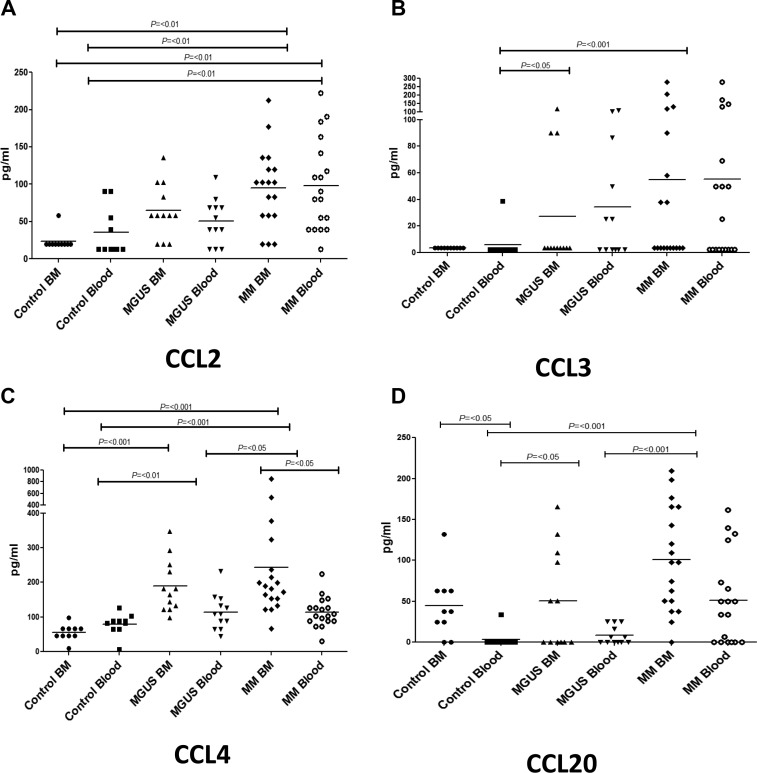
Plasma cytokine levels (pg/ml) of CCR4-related ligands were measured in bone marrow (BM) and blood in MM, MGUS and control patients by multiplex bead analysis (**A**) CCL2 (**B**) CCL3 (**C**) CCL4 (**D**) CCL20. Data were analysed using a Kruskal Wallis where *** = *p* < 0.001, ** = *P* < 0.01, * = *P* < 0.05.

We next went on to examine the expression level of chemokines which are major ligands for CCR4, a chemokine receptor found predominantly on T_H_2 cells. Luminex analysis was used to determine the levels of CCL2 (MCP-1), CCL3 (MIP-1α), CCL4 (MIP-1β) and CCL20 (MIP-3α) in serum samples from blood and bone marrow (Figure 2A–2D).

Within healthy controls the concentration of all the chemokines was found to be low and broadly similar between the blood and marrow compartments. Specifically, CCL2 and CCL3 were undetectable in marrow with very low levels being found within blood. CCL4 was measurable at low level in both marrow and blood whereas CCL20 was found at slightly higher level within the marrow and was not detectable in blood.

Remarkably, the expression patterns of CCL2, CCL3, CCL4 and CCL20 were very similar in the patient groups and showed substantially higher levels in both the blood and bone marrow compared to the control group. This increase in expression was pronounced, with relative increases of between 2 and 20 fold in MM patients compared to the healthy controls. A particularly striking feature was that chemokine levels increased sequentially during the clinical progression from healthy donors through to MGUS and finally MM.

### The C-X-C chemokines CXCL12 and CXCL8 are also progressively increased in blood and BM in patients with MGUS and MM

**Figure 3:**
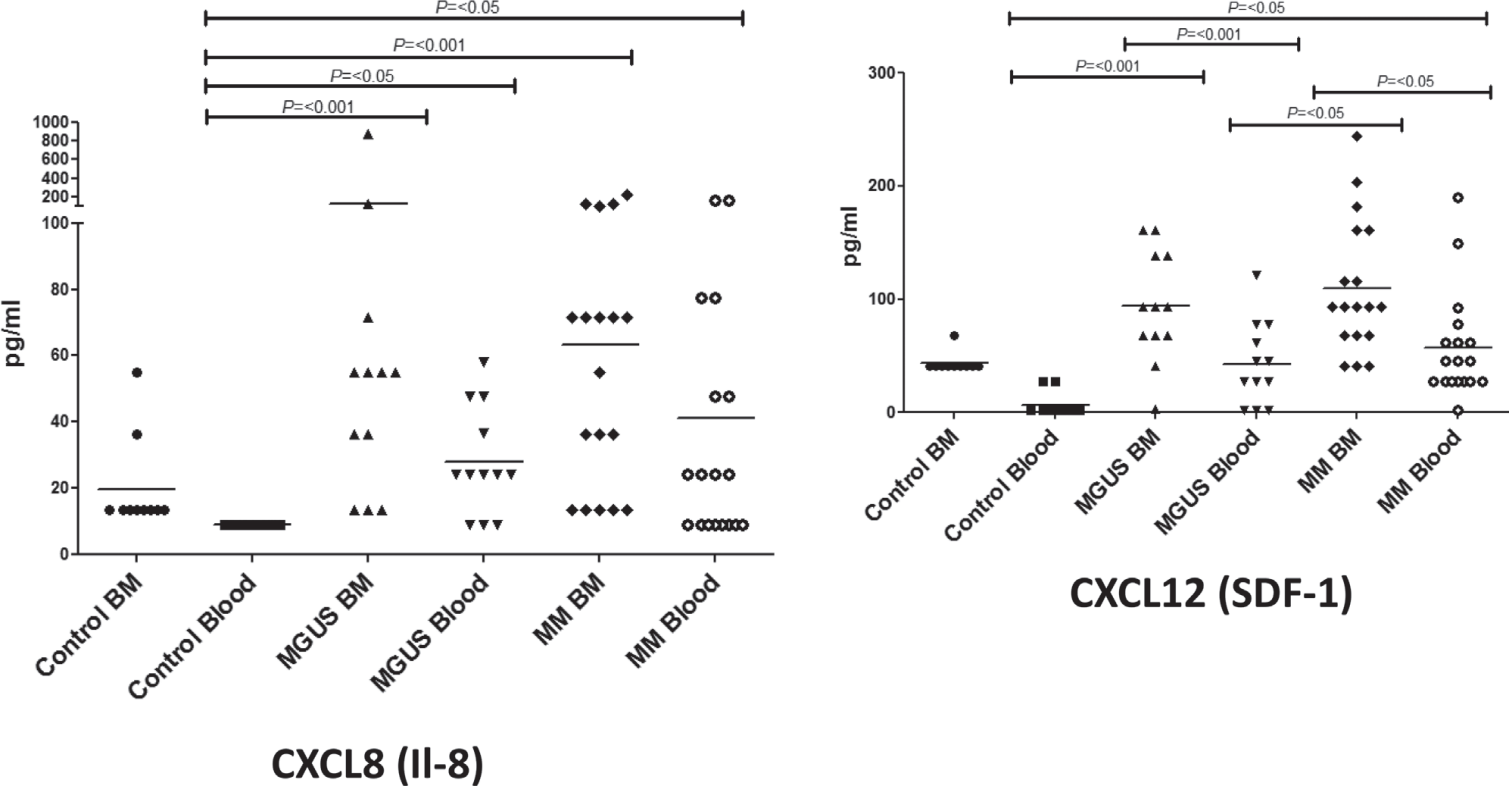
Plasma cytokine levels (pg/ml) of CXCL8 (**A**) and CXCL12 (**B**) were measured in bone marrow (BM) and blood in MM, MGUS and control patients by multiplex bead analysis. Data were analysed using a Kruskal Wallis where *** = *p* < 0.001, ** = *P* < 0.01, * = *P* < 0.05.

The expression of CXC chemokines such as CXCL12 (SDF-1) and CXCL8 (IL-8) has been reported to be modulated in patients with MM [31, 33–36]. CXCL12 is the ligand for CXCR4 whereas CXCL8 binds to the CXCR1 and CXCR2 receptors. We therefore studied the expression of these molecules in the blood and bone marrow within our study group (Figure 3A and 3B). Very marked changes were observed in the expression pattern between the control and patient groups. CXCL8 levels were very low in control group and only small amounts of CXCL12 were observed in the bone marrow of healthy subjects. In contrast, the expression of both molecules was increased in the patient group with the highest levels being observed in with the bone marrow of patients with myeloma (Figure 3A and 3B).

### The pattern of distribution of CXCR3+ T cells between blood and bone marrow is altered in patients with multiple myeloma

In view of the clear alteration in the pattern of distribution of the chemokine ligands for CXCR3 and CCR4 within our patient groups, we then went on to study the expression of these chemokine receptors on T cells in blood and bone marrow. Unfortunately it was not possible to analyse T cells within bone marrow samples from patients with MGUS due to lack of clinical samples.

**Figure 4:**
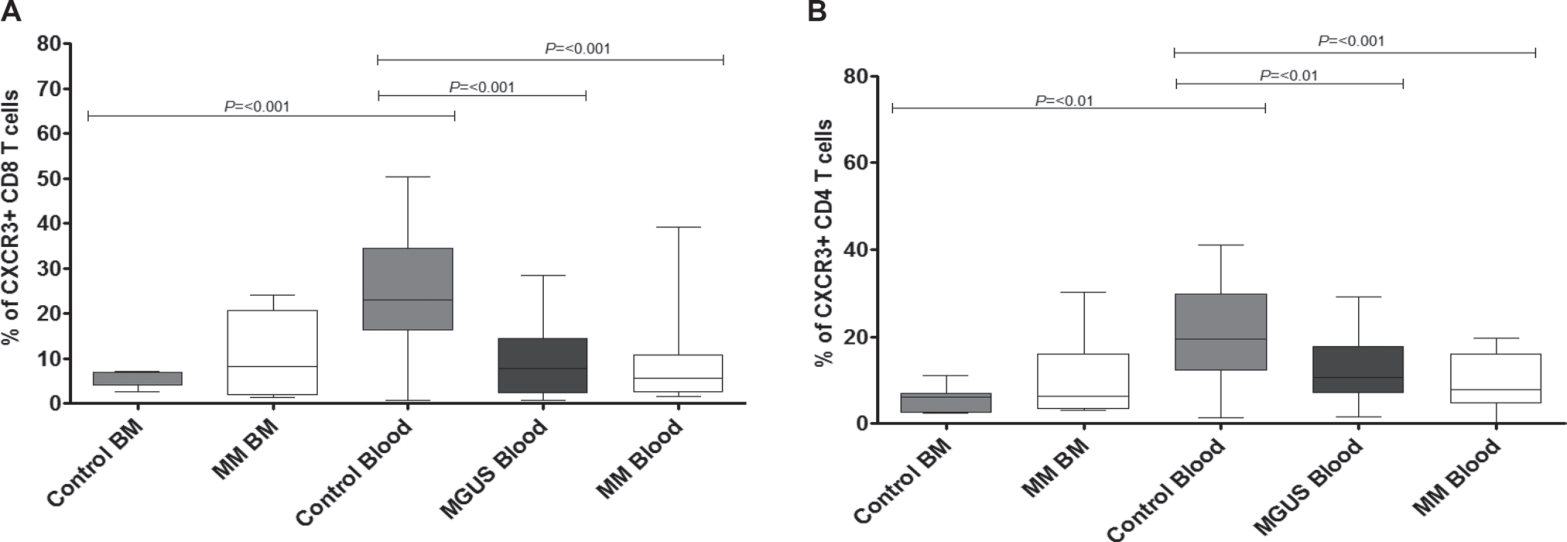
Percentage expression of CXCR3 on global CD8 T cells (**A**) and global CD4 T cells (**B**) in bone marrow (BM) and blood in MM, MGUS (blood only available) and control patients by flow cytometry. Data were analysed using a Kruskal Wallis where *** = *p* < 0.001, ** = *p* < 0.01, *p < 0.05.

CXCR3 expression is strongly correlated with a functional T_H_1 phenotype and in healthy control subjects the receptor was expressed on approximately 20–30% of both CD4+ and CD8+ T cells within blood. However, CXCR3+ T cells were relatively excluded from marrow where expression was seen on less than 5% of both populations. A different pattern was observed within the patient group. Expression of CXCR3 on T cells was reduced to approximately 10% within the blood of the patient group and a similar level of expression was observed within bone marrow. As such, the pattern of distribution of CXCR3+ T cells between blood and marrow, which is normally much higher in blood, was reversed in the patient groups (Figure 4A, CD8+ T cells; Figure 4B, CD4+ T cells).

### The expression of CCR4 is markedly increased on CD8 and CD4 T cells within the bone marrow of patients with myeloma

CCR4 expression is observed on the majority of T_H_2 lymphocytes and we next analysed the frequency of CCR4+ expression on CD8+ and CD4+ T cells within the study groups.

**Figure 5:**
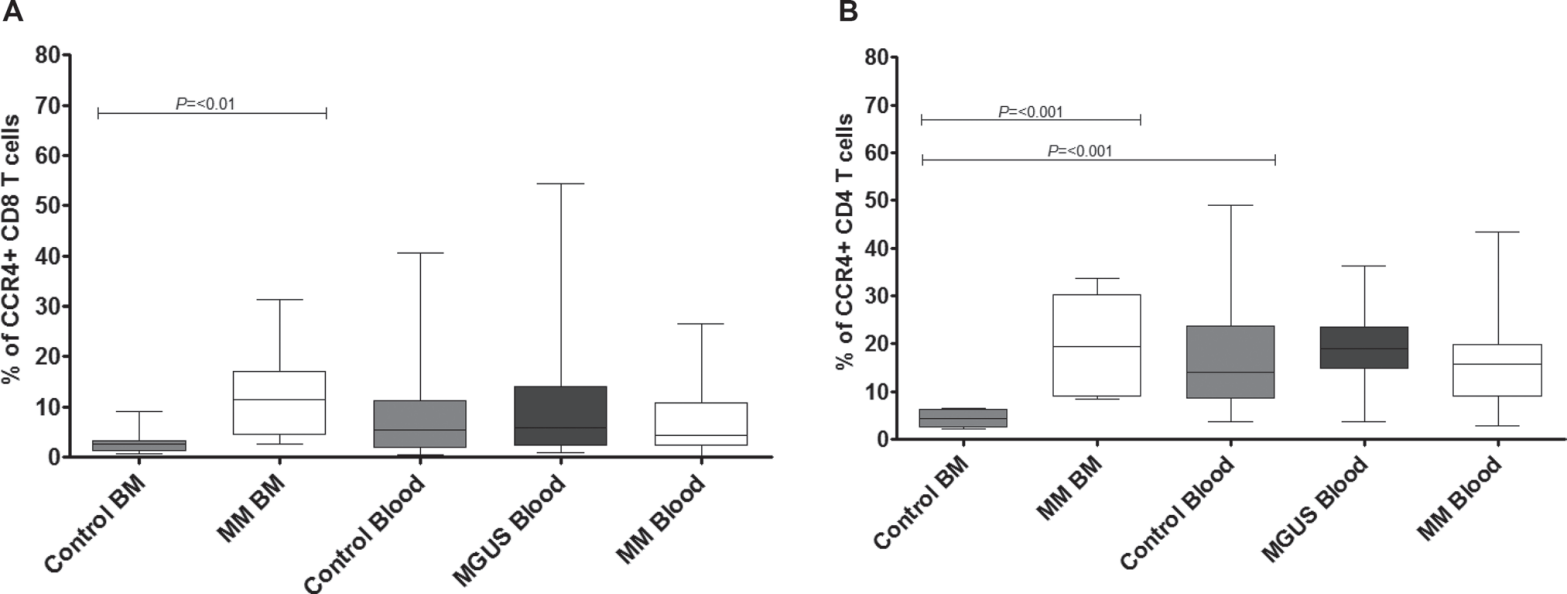
Percentage expression of CCR4 on global CD8 T cells (**A**) and global CD4 T cells (**B**) in bone marrow (BM) and blood in MM, MGUS (blood only available) and control patients by flow cytometry. Data were analysed using a Kruskal Wallis where *** = *p* < 0.001, ** = *p* < 0.01, *p < 0.05.

In control subjects there was a markedly higher expression of CCR4+ T-cells within the blood compared to the bone marrow (Figure 5A and 5B). Indeed, expression of this marker on T cells within marrow was low and represented a mean of 4.4% of the total population. In contrast the frequency of CCR4-expressing CD4+ T cells in BM was substantially increased in patients with myeloma to an average expression of 20% of the CD4+ T cell pool (**p = < 0.01). This led to a clear and marked reversal of the normal distribution ratio of cells between blood and bone marrow (Figure 5B) indicating that paraproteinaemia is associated with relative accumulation of T_H_2 T cells within the bone marrow microenvironment.

Overall it can be seen that T cells which express CCR4 or CXCR3 are strongly excluded from bone marrow within healthy individuals, whereas in the patient group this pattern is lost and a relative equivalence between compartments is observed due to an increased proportion of cells within marrow and fewer cells retained with peripheral blood.

### The percentage of CD4+ T_h_17 cells is also increased within the bone marrow in patients with multiple myeloma

**Figure 6:**
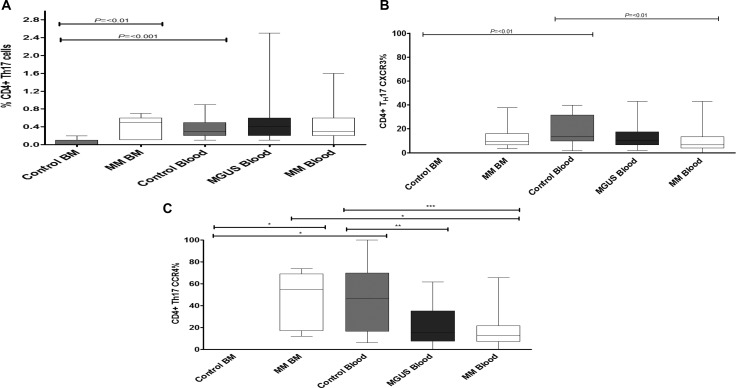
The frequency of CD4+ T_H_17 cells (**A**) was measured in both control individuals and MM and MGUS patients. The percentage expression of CXCR3 (**B**) and CCR4 (**C**) was then determined on the CD4+ T_H_17 cells within these compartments. Statistical analysis was by Kruskal Wallis where *** = *p* < 0.001, ** = *p* < 0.01, * *p* < 0.05

The potential importance of CD4+ T_H_17 cells in patients with multiple myeloma is an area of considerable interest and an increase in the frequency of such cells within the bone marrow has been reported [10, 13, 24, 25]. Within the control group T_H_17 cells comprised 0.3% of the CD4+ T cell pool but were almost completely excluded from bone marrow (*p* = 0.0002). In contrast there was an 8 fold increase in the frequency of such cells in the marrow of patients with myeloma such that they expanded to represent 0.45% of the lymphoid pool (*p* = 0.0041) (Figure 6A). In view of the increased proportion of T_H_17 cells within the bone marrow of the patient group we then went on to determine the potential role of CXCR3 and CCR4 in mediating this recruitment [37, 38]. CXCR3 and CCR4 expression on T_H_17 cells was indeed reduced in the blood of the MM group but increased within MM bone marrow (Figure 6B and 6C for CXCR3 and CCR4 respectively).

## DISCUSSION

MGUS and myeloma are associated with the accumulation of malignant plasma cells within the bone marrow and as plasma cells and T cell subsets make numerous molecular interactions it is likely that the T cell response plays an important role in determining the natural history of the disease. This could act potentially to either support the growth of the malignant clone or to suppress plasma cell growth through tumour surveillance. Many alterations of the T cell repertoire in blood and bone marrow have been reported in patients with multiple myeloma. The physiological basis for these is poorly understood but it is possible that they represent potentially interesting opportunities for therapeutic intervention.

We studied the level of expression of inflammatory chemokines in the blood and bone marrow of patients with MGUS and MM and focussed on chemokines that are major determinants of the migration of the T_H_1 and T_H_2 T cell subsets, for which CXCR3 and CCR4 are characteristic and relatively specific markers. Elevated levels of several chemokines have previously been documented in MM and are thought to play a role in promoting MM proliferation and survival [27–29, 31, 32, 34, 36, 39, 40]. The source of these chemokines appears to derive from both the myeloma plasma cells and associated stromal cells [34, 36, 40, 41]. As such, CXCL9 [42], CXCL12 [28] and CCL3 [43] have been shown to be produced by myeloma plasma cells whereas stromal cells have been shown to produce a range of chemokines, including CCL2, CCL7, CCL8 and CCL13 from osteoclasts [44], CXCL8, CXCL11, CXCL12 and CCL2 from endothelial cells and bone marrow fibroblasts producing CXCL12 [28]. The CXCR 4/CXCR7/ (SDF) axis has been shown to be important in the recruitment of monocytic lineage cells and increased levels of CXCL12 are believed to promote the differentiation of M2 macrophages [28, 30, 27, 32].

Chemokine expression levels were very low in the normal bone marrow and indeed CCL2, CCL3, CXCL9 and CXCL10 were completely undetectable. This is likely to reflect the role of marrow as both a primary and secondary lymphoid organ where homeostatic immune interactions predominate over inflammatory responses. A low but detectable level of several chemokines was observed within blood, which may reflect a degree of systemic inflammation within peripheral tissue, or physiological establishment of a marrow-vascular chemotactic gradient. It it noteworthy that high CXCL9 levels have recently been associated with poor clinical outcome in patients with myeloma [42].

In contrast chemokine expression was substantially increased in the bone marrow of the patient group. The magnitude of this effect was considerable with the most marked effects seen for CCL4 (CCR4 ligand) and CXCL9 (CXCR3 ligand), which were increased by 4 and 6 fold respectively in the bone marrow of patients with myeloma compared to controls. Li et al have also observed increased levels of CCL2 and CCL3 within the bone marrow of patients with myeloma but did not see an increased levels of CCL4 or CCL5 in their cohort [45]. These changes led to a marked reversal of the normal gradient of chemokine expression between blood and bone marrow in the patient groups. Specifically, whilst chemokine expression levels are normally higher in the vascular system, within the patient group they were either comparable between blood and marrow, or as in the case for CCL4, CCL20 and CXCL10, completely reversed such that chemokine levels were increased within marrow.

We next went on to investigate how these altered chemokine gradients might influence the distribution of major T cell subsets in patients with paraproteinaemia. T_H_1 cells are characterised by expression of CXCR3 whilst the great majority of T_H_2 cells express CCR4, and we therefore took these as markers of the two major functional subsets of T cells within the lymphoid system [46]. Again the pattern of distribution within the control group was interesting in that the proportion of cells expressing the chemokine receptor was low within the bone marrow, typically less than 5% of the lymphoid pool. In contrast, a high percentage of T cells expressed these markers in peripheral blood in keeping with many previous reports.

The pattern of distribution of CXCR3+ and CCR4+ T cells was markedly altered in patients with paraproteinaemia and showed a characteristic pattern. In almost all cases the percentage of chemokine receptor-positive cells was reduced in the blood and simultaneously increased in the bone marrow. Some of these alterations were striking with the proportion of bone marrow CD4 and CD8+ T cells which expressed CCR4 being increased from 4.4% and 3% respectively in the control group to 20% and 12% in patients with myeloma. A greater than two fold increase in CXCR3 expression on bone marrow T cells was also observed within the patient group. CXCR3 and CCR4 can be expressed on both central memory and effector memory T cells and it will be of interest in future work to define both the differential trafficking of discrete CD8 T cell memory subsets and the relation of this to expression of major transcriptional regulators..

Negligible numbers of T_H_17 cells were detected within the bone marrow from control subjects but were markedly increased in the bone marrow of myeloma patients in keeping with previous studies [10, 13, 25]. However, this population still represented less than 1% of the total CD4+ T cell population. The relative frequency of T_H_17 cells in blood was not altered and a comparable recruitment of these cells to solid tumours has also been reported [23]. T_H_17 cells may play a particularly important role in the development of clinical complications of paraproteinaemia and have been linked to problems such as bone disease. T_H_17 cells can express a wide range of chemokine receptors [34, 35] and in our study we were able to examine the expression of CXCR3 and CCR4. Our findings were very similar to those observed for the major CD4 and CD8 T cell subsets.

Several studies have demonstrated alterations in T regulatory cells in marrow samples from patients with paraproteinaemia, although there remains considerable controversy [12]. We did not examine the T regulatory subset but as these cells often express CCR4 the pattern of chemokine expression that we observed could certainly act as a mechanism to mediate their recruitment.

One limitation of our study is that we were not able to ascertain the absolute number of T cells within the different tissue compartments and so it is uncertain how proportional changes in T cell subsets reflect absolute changes in T cell distribution. Increased numbers of bone marrow T cells have been reported in MGUS and myeloma, and significant T cell infiltration is a common feature on diagnostic bone marrow aspirates and trephines [4]. In addition it should be noted that the increased proportion of T cells bearing chemokine receptors within marrow might potentially reflect increased local proliferation as well as preferential trafficking.

The relative importance of the individual chemokines and their ligands will need further investigation. It was noteworthy, however, that the frequency of CCR4+ T cells was substantially greater than the CXCR3+ population, suggesting that CCR4-binding chemokines may have a predominant influence on recruitment and retention of T cells to marrow in patients with myeloma. In addition we were not able to examine all of the chemokine receptors expressed by T cells and the level of their related chemokine ligands. Many of the chemokines that bind to CCR4 can also interact with additional chemokine receptors (e.g. CCR2, CCR5). Certainly we were able to observe that SDF-1, which binds to CXCR4, and CXCL8 (IL-8) which is a ligand for CXCR1/CXCR2, are also significantly elevated in myeloma. As such the role of the chemokine system in controlling the distribution of T cells within the lymphoid compartment in patients with myeloma is likely to be highly complex. Ponzetta et al have recently shown that administration of myeloma cells to immunodeficient mice leads to an increase in CXCL9 and CXCL10 within the murine bone marrow, supporting a direct role for tumour cells in the generation of inflammatory mediators [47]. In addition they also observed increased levels of CXCL10 within the bone marrow of patients with multiple myeloma and this was correlated with a decrease in the number of CXCR3+ NK cells within blood.

**Figure 7:**
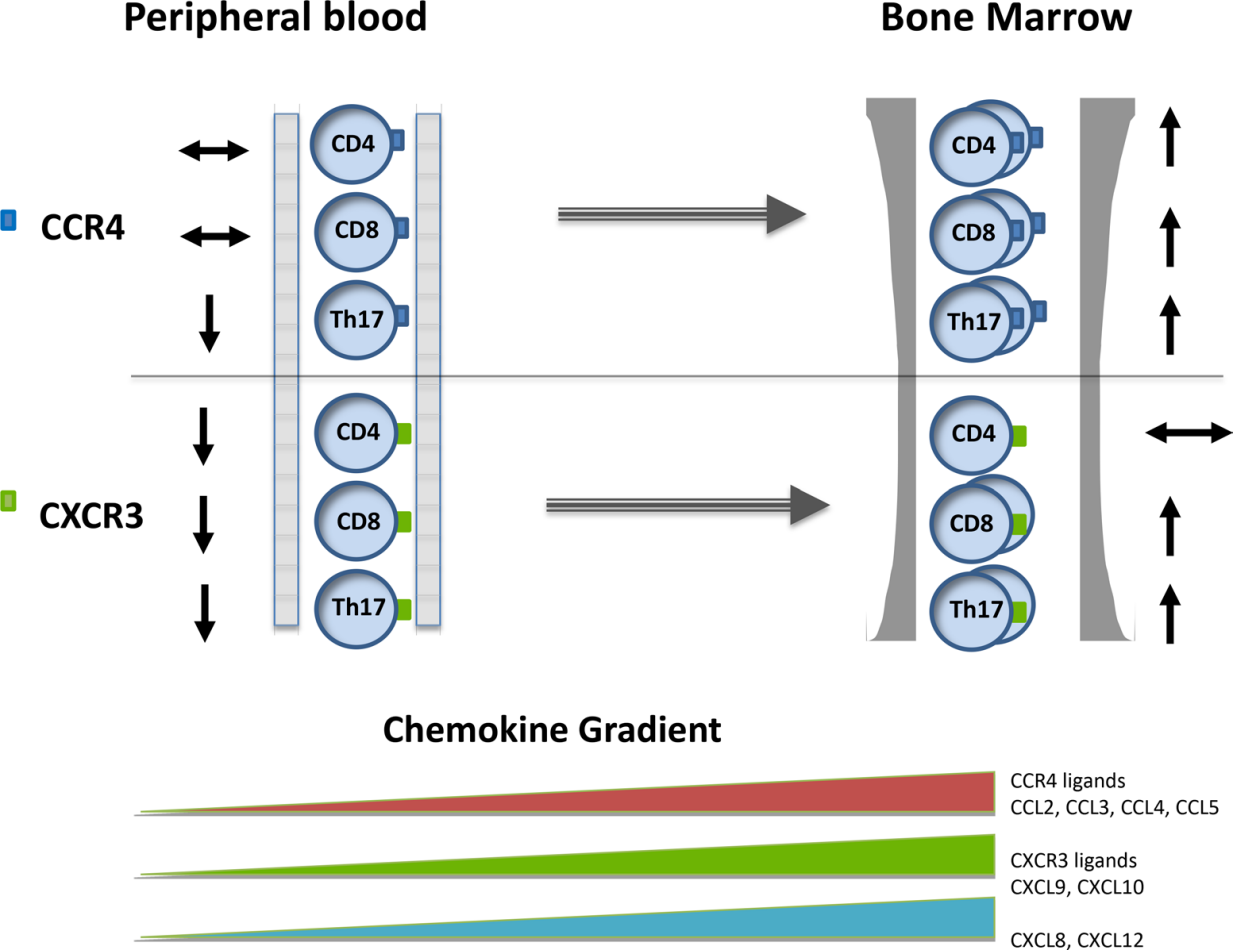
The pattern of chemokine concentration and CXCR3+ and CCR4+ expression on T cell subsets within the blood and bone marrow in patients with paraproteinaemia

Our findings clearly indicate the presence of an ‘inflammatory environment’ in the bone marrow of patients with multiple myeloma and we hypothesise that these chemokine gradients serve to drive T cell distribution as illustrated in Figure 7 although further work is needed to confirm this model. It will now be important to examine the relative roles of the neoplastic plasma cell and the tumour microenvironment in mediating this effect. These altered chemokine gradients may play a major role in the redistribution of T cell subsets in patients with paraproteinaemia. Importantly this effect occurs early in disease pathogenesis as it is clearly observed in the MGUS stage before the development of clinical complications. It is interesting to speculate on the potential clinical importance of the altered T cell subset distribution in relation to the clinical features of paraproteinemia. Increased susceptibility to infection is a major feature of this disorder and impairment of the adaptive immune response has been shown in several studies. In contrast, this may represent early evidence of a tumour-specific immune response and, indeed, only 1% of patients with MGUS progress to myeloma each year. A role for CXCL10 in the control of myeloma cell growth has indeed been observed [48]. However there is also considerable evidence that tumours induce an inflammatory response within their environment as a mechanism to support their survival and invasion. As such, anti-inflammatory drugs are being assessed for their potential therapeutic value in a range of diseases. Indeed, bone marrow levels of CCL3 and CCL20 have been shown to be higher in myeloma patients with increased levels of bone disease [49]. Recently Jak-2 inhibition with ruxolitinib and fedratinib have been shown to silence T helper cell cytokine production and reduce T regulatory cells [50]. Interventions that are able to suppress the elevated chemokine levels observed in patients with myeloma may therefore have potential beneficial effects and would be worthy of consideration as novel therapeutic agents [51].

## MATERIALS AND METHODS

### Patients

Patients with MM and MGUS were recruited from specialist clinics at The Royal Wolverhampton Hospitals and Heart of England NHS Foundation Trusts. The study received approval from the Local Ethics Committees and informed written consent was obtained in all cases. Bone marrow aspirates (~1 ml) and peripheral blood (~5 mL) were obtained from patients at various stages of disease and mononuclear cells were purified by density centrifugation using Lymphoprep (Nycomed, Oslo, Norway). Plasma was collected after cell separation and snap frozen at −80°C. For studies of chemokine levels we obtained samples from 18 MM patients (12 at diagnosis, 3 at relapse, 1 refractory disease and 2 post-treatment) and 12 MGUS patients using paired bone marrow and blood samples.

PBMC were available from 37 MM patients (22 at diagnosis, 4 at relapse, 2 in ‘plateau phase’ and 9 asymptomatic patients) and 23 MGUS patients, and were used for examination of T cell subsets. Paired bone marrow and blood samples were also available from 9 MM patients for comparative study of T cell profiles. 10 patients undergoing thoracotomy surgery were recruited as a control group, from which bone marrow was available on 7 subjects. None of these patients had bone marrow disease or were undergoing treatment for significant medical conditions. A section of rib was obtained during surgery and the bone marrow flushed through using RPMI1640 with a needle and syringe. Mononuclear cells and plasma were harvested as above.

### Luminex methodology

Multiplex bead analysis was performed on patient plasma samples using the Procarta Cytokine Assay Kit (Affymetrix, High Wycombe, UK) for a range of cytokines and chemokines including CXCL8 (IL-8), CXCL9 (MIG), CXCL10 (IP-10), CXCL11 (ITAC), CXCL12 (SDF-1), CCL2 (MCP-1), CCL3 (MIP-1α), CCL4 (MIP-1β), CCL5 (RANTES) and CCL20 (MIP-3α). Measurements were performed as per manufacturer’s instructions. Briefly, plasma samples, diluted with an equal volume of PBS, were incubated with monoclonal antibody-coated capture beads for 1 hour at 20°C. Washed beads were further incubated with biotin-labelled polyclonal anti-human cytokine antibody for 30 minutes followed by streptavidin-phycoerythrin for 30 minutes. Samples were analysed with a bioassay analyser (model 100; Luminex, Austin, USA). Standard curves of known concentrations of human antigen (Affymetrix, High Wycombe, UK) were used to convert fluorescence units to cytokine concentration (pg/ml). Results were calculated from standard curves prepared on each plate.

### Intracellular cytokine analysis

Mononuclear cells were stimulated with PMA and ionomycin for six hours in the presence of ‘Golgi stop’ (eBioscience, San Diego, CA) as described previously [33]. Briefly, cells were stained with surface antibodies CD8 Amcyan (BD Biosciences. Oxford, UK), CD3 APC-Cy7 (Cambridge Biosciences, Cambridge, UK), CCR4 PE-Cy7 (BD Biosciences, Oxford, UK) and CXCR3 PE (BD Biosciences. Oxford, UK) for 20 mins at 4°C. Samples were washed, re-suspended in RPMI with 10% human serum (TCS Biosciences, Buckingham, UK) and then stimulated with PMA and ionomycin for six hours in the presence of ‘Golgi stop’. Cells were washed, stained with a dead cell exclusion dye (Invitrogen, Oregon, USA) at 4°C for 20 mins and then washed again before fixation with 2% paraformaldehyde at room temperature for 10–15 mins. After further washing, cells were permeabilised with 0.5% saponin for 5 minutes followed by incubation with the intracellular antibodies IL-17A (BioLegend, London, UK) (2.5 ml), IFN-γ (BioLegend, London, UK) (0.5 ml) and isotype controls for 30 mins at room temperature. Samples were washed and analysed by flow cytometry using on LSR II flow cytometry. To define Th17 cells we measured the secretion of IL-17 using intracellular staining and FACS analysis.

### Statistical analysis

A Kruskal-Wallis test was used to assess the level of significance between plasma proteins produced by MM, MGUS and control patients. This was performed using Prism 5 for Windows (GraphPad, San Diego, CA). A Kruskal-Wallis test was also used to compare chemokine expression on T cells between peripheral blood and bone marrow mononuclear cells from MM, MGUS and aged-matched controls. Mann-Witney *t-test* was used to assess the level of significance of the percentage of CD4 IL-17 cells and CD4 T regulatory cells between peripheral blood and bone marrow mononuclear cells from MM, MGUS and aged-matched controls and also to test the significance of CXCR3 and CCR4 expression on total CD4 and CD8 T cells.
